# Proposal of Potent Inhibitors for a Bacterial Cell Division Protein FtsZ: Molecular Simulations Based on Molecular Docking and ab Initio Molecular Orbital Calculations

**DOI:** 10.3390/antibiotics9120846

**Published:** 2020-11-26

**Authors:** Shohei Yamamoto, Ryosuke Saito, Shunya Nakamura, Haruki Sogawa, Pavel Karpov, Sergey Shulga, Yaroslav Blume, Noriyuki Kurita

**Affiliations:** 1 Department of Computer Science and Engineering, Toyohashi University of Technology, Tempaku-cho, Toyohashi, Aichi 441-8580, Japan; yamamoto@klab.cs.tut.ac.jp (S.Y.); saito@klab.cs.tut.ac.jp (R.S.); nakamura@klab.cs.tut.ac.jp (S.N.); sogawaharuki@gmail.com (H.S.); 2 Institute of Food Biotechnology and Genomics, National Academy of Sciences of Ukraine, 2a, Osypovskogo str., Kyiv-123, 04123 Kyiv, Ukraine; karpov@ifbg.org.ua (P.K.); Shulga5@i.ua (S.S.); blume.yaroslav@nas.gov.ua (Y.B.)

**Keywords:** FtsZ, *Mycobacterium tuberculosis*, Zantrin, inhibitors, fragment molecular orbital, protein–ligand docking, in silico drug design

## Abstract

The inhibition of a bacterial cell division protein, filamentous temperature-sensitive Z (FtsZ), prevents the reproduction of *Mycobacteria*. To propose potent inhibitors of FtsZ, the binding properties of FtsZ with various derivatives of Zantrin ZZ3 were investigated at an electronic level, using molecular simulations. We here employed protein–ligand docking, classical molecular mechanics (MM) optimizations, and ab initio fragment molecular orbital (FMO) calculations. Based on the specific interactions between FtsZ and the derivatives, as determined by FMO calculations, we proposed novel ligands, which can strongly bind to FtsZ and inhibit its aggregations. The introduction of a hydroxyl group into ZZ3 was found to enhance its binding affinity to FtsZ.

## 1. Introduction

Tuberculosis (TB) is one of the most widespread infectious diseases. It is caused by the bacillus *Mycobacterium tuberculosis* (Mtb). The 2017 report of the World Health Organization (WHO) has revealed that approximately 10.4 million people are diagnosed with TB each year, and approximately 1.7 million TB patients lose their lives [1,2]. To overcome this critical situation, WHO aims to establish technologies to accelerate the development of novel drugs, which are effective for the prevention of a severe TB epidemic.

Various kinds of drugs, including peptides, natural products, and other synthetic small molecules, have been developed and employed for the treatment of TB. However, there is a considerable risk of Mtb to develop excellent resistance to these drugs [3,4]. Particularly, multidrug-resistant TB (MDR-TB) is one with resistance to multiple drugs, and the number of MDR-TB cases has rapidly increased. In fact, WHO [2] reported 490,000 cases of MDR-TB resistance to rifampicin, one of the most effective TB drugs. Furthermore, approximately 9.5% of MDR-TB cases have been identified to be extra-drug resistant (XDR-TB) and they are untreatable by any drug. Accordingly, the development of effective anti-TB drugs that can target more conservative proteins is strongly required to prevent the occurrence of MDR-TB and XDR-TB [5]. In addition, TB should be completely treated within a short time because the prolonged treatment might cause MDR-TB. Therefore, the development of strong anti-TB drugs with a reduced treatment period is desired [6].

For developing new anti-TB drugs, a cytoskeletal protein, filamentous temperature-sensitive Z (FtsZ), has been considered as a target protein recently. Since FtsZ contributes greatly to the formation of cell walls during cell division, it is indispensable for cell division in bacteria. Accordingly, new drugs, which suppress the proliferation of Mtb by inhibiting the division of the Mtb cells, have been recently developed [7].

In fact, FtsZ proteins, which exist around bacterial cells, and a series of proteins called the Min family [8] control the cell divisions. A number of FtsZs form a cytokinetic ring (Z-ring) around the center of the cell, at the beginning of prokaryotic cell division. Subsequently, the Min proteins associate with the cell division using the Z-ring as a scaffold for binding [8]. As a result, a new cell wall is developed between the dividing cells. Finally, by closing the Z-ring, the cell is divided into two cells. Therefore, an inhibitor of the formation of the Z-ring composed of FtsZs is expected to efficiently suppress the proliferation of the Mtb cells. In addition, the inhibitor is likely going to exert a slight side effect because the amino acid sequence in the homologous site of Mtb FtsZ is significantly (from 35% to 99%) different from those in FtsZs of other bacteria in the human body [9].

About the mechanism of the FtsZ- and Min family-controlled Z-ring formation, there are many unresolved challenges. A recent in vitro study [5] revealed that there are several binding sites for inhibitor in FtsZ that are challenging to determine by experiments alone. Moreover, since the amino acid sequence in FtsZ is different for each bacterium, it is impossible to predict the binding site in FtsZ of Mtb from those of FtsZ of bacteria. Consequently, the addition of molecular simulations is necessary for the elucidation of the specific interactions between FtsZ of Mtb and its inhibitors.

In our previous molecular simulations [10,11], the specific interactions between FtsZ of Mtb and its inhibitors, the curcumin derivatives and Zantrins (Z3 and ZZ3), were investigated by molecular simulations, which were based on protein–ligand docking, classical molecular mechanics (MM), and ab initio fragment molecular orbital (FMO) calculations. Curcumin is a natural product present in the root of *Curcumae rhizoma*. Its derivatives have been widely used as conventional drugs to treat many diseases [12,13]. In fact, it was observed that curcumin could suppress bacterial cell proliferation by inhibiting the formation of the Z-ring [14].

Z3 was previously synthesized and investigated by Margalit et al. [7], while ZZ3 was developed based on Z3 [15]. These Zantrins inhibited the activity of GTPase of FtsZ and prevented the generation of the Z-ring. Their half-maximal inhibitory concentrations (IC_50_) were 24 (Z3) [7] and 12 (ZZ3) μM [15], indicating that the inhibitory effect of ZZ3 is higher than that of Z3. Our previous molecular simulations [11] determined the binding affinity between FtsZ and Z3/ZZ3 and explained the trend of their IC_50_ values. We specified the most preferred binding sites of Z3/ZZ3 in FtsZ and highlighted the key amino acid residues of FtsZ that contributed to its binding to Z3/ZZ3 at an electronic level. We also revealed why ZZ3 is more potent than Z3 against FtsZ and that ZZ3 is effective for inhibiting the aggregation of FtsZ.

**Figure 1:**
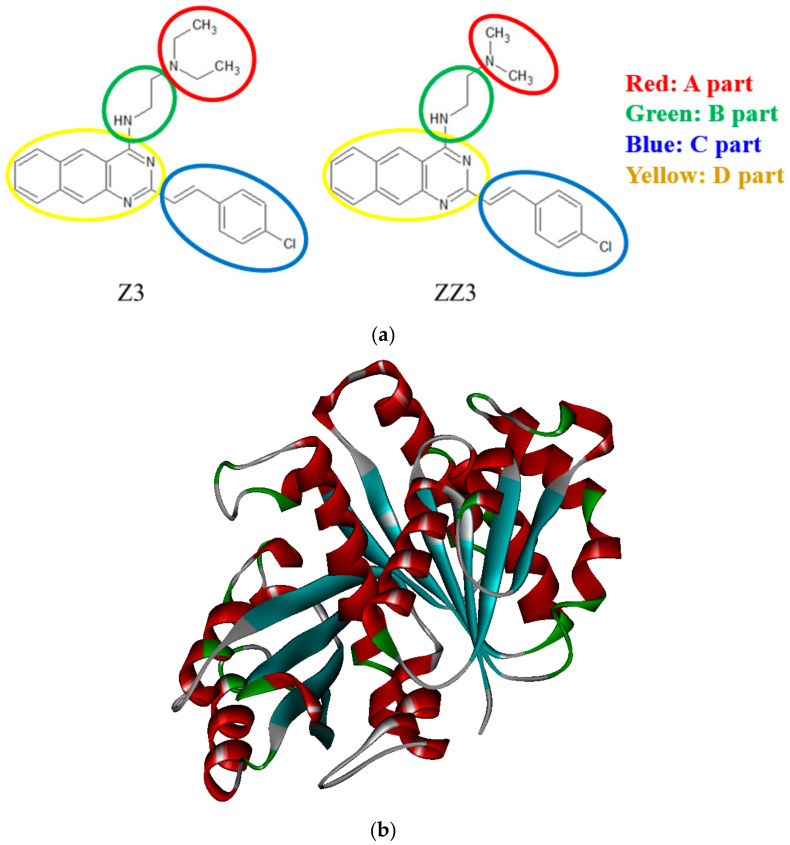
(**a**) Chemical structures and the definition of each part of Z3 and ZZ3, (**b**) a whole structure of Mtb FtsZ.

In this study, to propose additional potent inhibitors of FtsZ aggregation, we prepared many types of derivatives based on ZZ3 (Figure 1a) and investigated their binding conformations in FtsZ of Mtb (Figure 1b) by the molecular simulations, focused on the site containing the H6/H7 loop and H7 of FtsZ, because the site contributes to the FtsZ aggregation. Furthermore, the specific interactions between FtsZ and the prepared derivatives were precisely analyzed at an electronic level by ab initio FMO calculations. The results will be beneficial for proposing novel anti-TB drugs that can disrupt the aggregation of FtsZ.

## 2. Details of Molecular Simulations

### 2.1. Proposal of Novel ZZ3 Derivatives as Potent Inhibitors of FtsZ

To search for novel ZZ3 derivatives widely, we employed SwissBioisostere (the database of molecular replacements for ligand design) [16], and their chemical, pharmacokinetic, and toxic properties were verified on SwissADME (the website for predicting ADME parameters, pharmacokinetic properties, drug-likeness, and medicinal chemistry friendliness) [17] and PreADMET (a web-based application for predicting the ADME data and building a drug-like library by the in silico method) [18]. Among the ZZ3 derivatives produced by molecular replacements from SwissBioisostere, we selected some candidates with the desired pharmacokinetic and toxic properties and investigated their binding properties to determine those that can bind more strongly to FtsZ.

Initially, a series of ZZ3 derivatives were designed by replacing the A-part of ZZ3 (Figure 1a), using the molecular replacements obtained from the SwissBioisostere database [16]. In this database, structural information on 4.5 million molecular replacements and their information on biochemical assays are collected. The biochemical information is produced through the detection of matching molecular pairs and by mining the bioactivity data in the ChEMBL database.

Next, the pharmacokinetic and medicinal properties of the selected derivatives were verified by SwissADME [17]. The derivatives were screened by Lipinski’s rule of five (RO5) [19] and Veber’s rules for compounds: (1) molecular weight (MW), <500 Da; (2) number of rotatable bonds (RBs), <12; (3) number of H-bond acceptors (HBAs), <10; (4) number of H-bond donors (HBDs), <5; (5) octanol–water partition coefficient (LogP), <5; and (6) polar surface area (PSA), <140 Å^2^. In the present study, we defined an H-bond when the distance between H and Y atoms in the XH--Y interaction was shorter than 2 Å.

We also verified if these derivatives satisfied the required absorption, distribution, metabolism, excretion, and toxic conditions (the ADMET rule) through the PreADMET software [18]. The software can predict the values of physically relevant descriptors and pharmaceutically beneficial properties of the ZZ3 derivatives, and these values can be compared with the recommended values for ideal drugs. In selecting the beneficial derivatives, we considered the blood–brain barrier (BBB) penetration, the heterogeneous human epithelial colorectal adenocarcinoma cell lines (Caco-2), the human intestinal absorption (HIA), the plasma protein binding, the toxicity to mice and rats, and the inhibition risk of the human ether-a-go-go-related gene (hERG). In fact, only the derivatives that satisfied the following conditions were selected: BBB > 0.1, Caco-2 > 4, and HIA > 20%. The binding affinities of FtsZ with the selected ZZ3 derivatives were investigated by molecular simulations similar to our previous studies [10,11].

### 2.2. Constructions and Optimizations of the FtsZ + Derivative Complexes

In modeling the initial structure for FtsZ, we employed the X-ray crystal structure of the Mtb FtsZ + GDP complex (Protein Data Bank (PDB) ID: 1RQ7 [20]). This PDB structure contains two subunits of FtsZ (chain-A and chain-B), and only chain-A possesses the complete structure of the ligand-binding pocket. We, therefore, employed chain-A as the initial structure for FtsZ. As the information on the residues from Arg64 to Ala68 is missing in the PDB structure, these missing residues were completed by a protein modeling server, Iterative Threading Assembly Refinement (I-TASSER) [21,22]. Five candidate models were produced by I-TASSER, and the model-1 was selected because it obtained the best C-score (1.86). FtsZ contains one His-residue, and its protonated state was assigned based on the pKa value predicted by the PROPKA3.1 program [23,24]. Since the pKa value of the His-residue was >6, the residue was assigned to the Hip^+^ protonation state. The N- and C-termini of FtsZ were terminated by the acetyl and amine groups, respectively.

The structures of the ZZ3 derivatives were fully optimized in vacuo, utilizing the B3LYP/6-31G (d,p) method of the ab initio molecular orbital calculation program, Gaussian09 (G09) [25]. The charge distributions of the optimized structures were evaluated by the restrained electrostatic potential (RESP) [26] analysis of G09 through the HF/6-31G (d) method, and the results were employed as the charge parameters in the MM force fields of the ZZ3 derivatives. Notably, these charge distributions are essential for the docking simulations of the ZZ3 derivatives to FtsZ, as well as the MM optimizations of the FtsZ + derivative complexes, because these charges precisely describe the electrostatic interactions between FtsZ and the ZZ3 derivatives.

In our previous molecular simulations [10,11] for the FtsZ complexes with curcumin derivatives (Z3 and ZZ3), we conducted the two steps of docking simulations to widely search for the preferred ligand-binding site because the most preferred site in FtsZ (possessing several ligand-binding sites) is unclear. The results [11] revealed that both Z3 and ZZ3 preferentially bind to the sites containing the H6/H7 loop and H7 and not to the GTP/GDP binding site of FtsZ, implying that Z3 and ZZ3 could effectively inhibit the aggregation of FtsZ by changing the conformation of the H6/H7 loop that facilitates the aggregation. Accordingly, the ZZ3 derivatives were docked to the same docking site of ZZ3 in FtsZ to investigate the effect of the replacement of a-part of ZZ3 on its binding to FtsZ.

In this docking simulation by the Autodock4 program [27], the grid box of docking was set as the center of gravity for ZZ3 in the optimized structure of the FtsZ + ZZ3 complex, and the size of the grid box was set to approximately two times the size of the long axis of the derivative (30 × 30 × 30 Å^3^). The number of candidate poses was 250, and they were grouped into several clusters based on the distance between them. Among various clusters that were generated, the cluster with the largest number of poses was selected, as obtained by the docking, and the representative structure of the cluster was employed in the subsequent MM and FMO calculations.

To obtain stable structures for the FtsZ + derivative complexes, the representative structure of the cluster obtained by the docking simulations was employed, and its potential energy was minimized in explicit water molecules by the classical MM method without a periodic boundary condition. As in the same manner of our previous study [11], about 1800 water molecules existing within 8 Å from the surface of the complex were explicitly considered in the MM method. We employed the MM and molecular dynamics simulation program AMBER12 [28], in which the AMBERFF99-SBLIN force field [29], the TIP3P model [30], and the general AMBER force field (GAFF) [31] were assigned to FtsZ residues, the water molecules, and the ZZ3 derivatives, respectively. The criterion for the convergence of the potential energy minimization of MM was set to 0.0001 kcal/mol/Å.

### 2.3. FMO Calculations for the FtsZ + Derivative Complexes

To elucidate the specific interactions and binding affinities between FtsZ residues and the ZZ3 derivatives, we investigated the electronic properties of the FtsZ + derivative complexes in explicit waters by the ab initio FMO method [32]. To consider the effect of the hydrating water molecules on the interactions between FtsZ and the derivatives, the water molecules existing within 10 Å of the derivative were explicitly considered and mean-field effects were not considered. The structure of the complex obtained by the MM method was employed for the ab initio FMO calculation.

In the FMO calculations, the ab initio MP2/6-31G method [33,34] of the FMO calculation program, ABINIT-MP Ver. 6.0 [35], was employed. Each amino acid residue of FtsZ, the ZZ3 derivative, and each water molecule were assigned to a fragment in the present FMO calculations, because this fragmentation enabled the analysis of the interactions between each amino acid residue of FtsZ and the derivative affected by the solvating water molecules. In addition, to highlight the essential FtsZ residues for strong binding to the ZZ3 derivatives, we analyzed the inter-fragment interaction energies (IFIEs) [36] obtained by the FMO calculations.

Furthermore, to predict the binding affinity between FtsZ and the ZZ3 derivative, the binding energy (BE) of FtsZ with the derivative was estimated from the total energies (TEs) of the component structures through the following equation:

BE = TE (complex + water) − TE (FtsZ + water) − TE (derivative + water) + TE (water)

In the above calculation, we employed the structure of the solvated complex, which was obtained by the MM method. From the structure, we picked up the structures of FtsZ + water, derivative + water, and only water, and evaluated the TEs of these structures as well as the solvated complex by ab initio FMO method.

In the present study, we did not consider the effect of entropy on the binding affinity, because the statistical ensembles for the solvated FtsZ + ZZ3-derivative complexes are very time consuming and not practicable by the ab initio FMO method, and because the entropic effect is likely to be not so different for each of the derivatives, which have almost the same chemical structures. Accordingly, we investigated binding energies between FtsZ and the derivatives by use of the ab initio FMO method and predicted the binding affinity, under the assumption that the entropic effect is the same for the ZZ3 derivatives.

## 3. Results and Discussion

### 3.1. Binding Properties between FtsZ and the ZZ3 Derivatives by Replacing A-Part

In our previous study [11], the specific interactions between FtsZ and Zantrins Z3 and ZZ3 were investigated by molecular simulations. The results were consistent with the experimental binding affinity trends of Z3/ZZ3 to FtsZ [7,15]. It was also revealed that ZZ3 could bind more strongly to the residues that were around the ligand-binding pocket of FtsZ, because the size of the A-part of ZZ3 was smaller than that of Z3 (Figure 1a). Accordingly, we first attempted the proposal of more potent inhibitors of FtsZ by adjusting the A-part of ZZ3 to fit into the ligand-binding pocket of FtsZ (Figure 1b).

**Figure 2:**
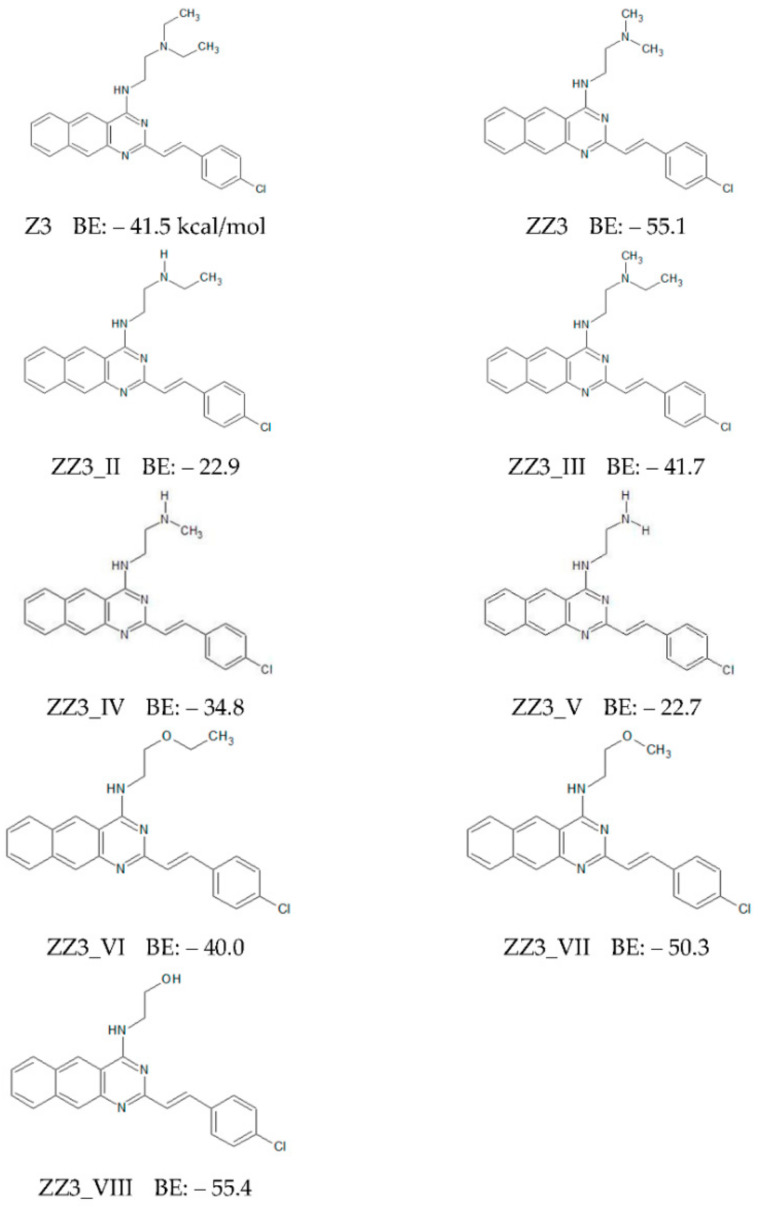
Chemical structures of Z3, ZZ3 and its derivatives with replacing A-part, and their binding energies (BE: kcal/mol) to FtsZ evaluated by ab initio FMO method.

First, we replaced the A-part of ZZ3 with other groups with smaller sizes and produced its derivatives (ZZ3_II, ZZ3_III, ZZ3_IV, and ZZ3_V), and their chemical structures are shown in Figure 2. The A-parts of these derivatives are expected to enter the ligand-binding pocket of FtsZ because they are smaller than that of ZZ3. In addition, considering that the nitrogen atom included in the A-part of ZZ3 strongly interacts with the OH group of the Ser176 side chain [11], the A-part was replaced by other groups, including an oxygen atom, to enhance the electrostatic interactions between the A-part of ZZ3 and the FtsZ residues, such as Ser176. These derivatives were named ZZ3_VI, ZZ3_VII, and ZZ3_VIII, and their chemical structures and BEs to FtsZ are shown in Figure 2.

Before performing docking simulations for these ZZ3 derivatives, their chemical and pharmacokinetic properties (ADMET) were verified on SwissADME [17] and by the PreADMET [18] software. As listed in Table 1, all the ZZ3 derivatives, as well as Z3 and ZZ3, satisfied Lipinski’s rules of five [19] and Veber’s rule. Therefore, it was confirmed that the ZZ3 derivatives possessed chemical properties suitable for pharmaceutical agents.

Furthermore, as listed in Table 2, all the ZZ3 derivatives satisfied the conditions for ideal drugs and could be ingested and absorbed by the human body. With regard to the toxicity, negative toxicity was exhibited in a carcino-rat, whereas positive toxicity in a carcino-mouse. The estimated inhibition risk of hERG of the derivatives was moderate. Therefore, the derivatives could be possibly toxic to some species.

The ZZ3 derivatives were docked to the ZZ3 binding site in FtsZ, and the potential energies of the obtained structures of the FtsZ + derivative complexes were minimized in explicit waters by the classic AMBER-MM method. The BEs between FtsZ and each ZZ3 derivative were evaluated by the ab initio FMO method. As shown in Figure 2, among the seven derivatives, only ZZ3_VIII possessed similar BE as that of pristine ZZ3, while BEs of the other derivatives were smaller. Accordingly, the replacement of the A-part of ZZ3 (Figure 1a) did not exert any significant effect on enhancing the binding interaction between FtsZ and ZZ3.

**Figure 3:**
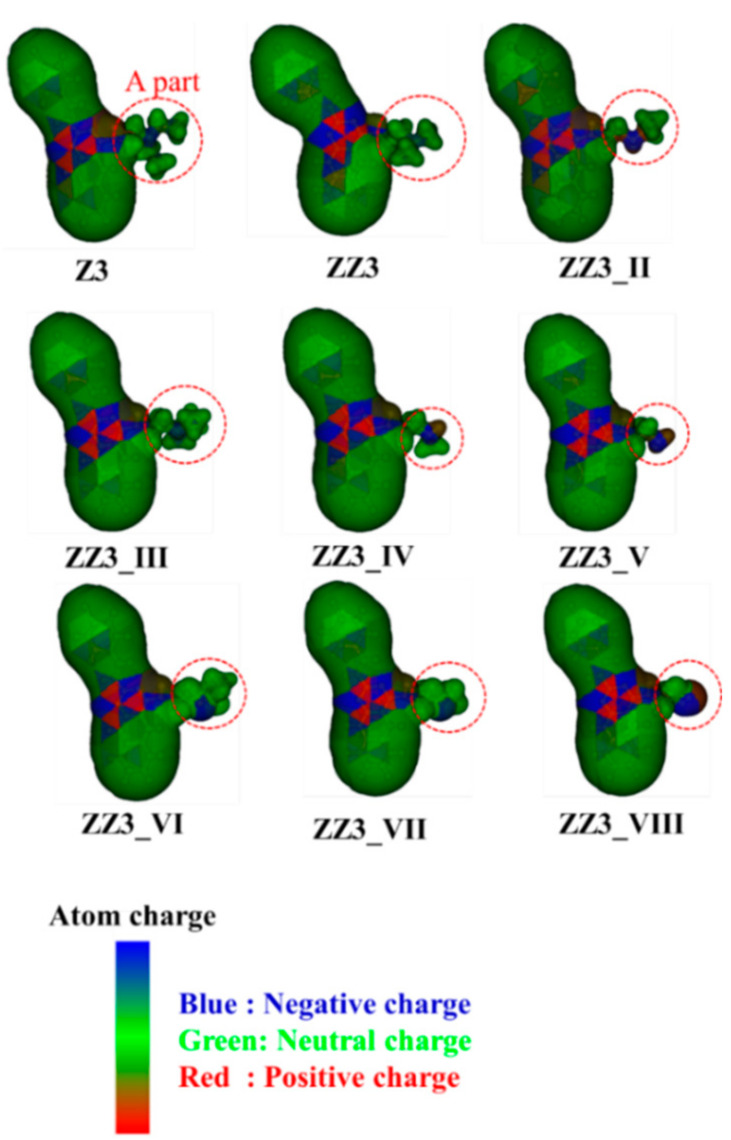
Charge distributions for Z3 and ZZ3 and its derivatives with replacing the A-part of ZZ3. The distributions are analyzed by RESP method of G09.

To elucidate the reason for this result, we investigated the charge distributions of the ZZ3 derivatives. Figure 3 reveals that the charge polarization around the nitrogen atom included in the A-part increased due to the reduction of its (A-part) size. Therefore, the electrostatic interactions between the A-part and the charged amino acid residues of FtsZ were expected to be enhanced by the reduction of the size of the A-part.

As shown in Figure S1 of the Supplementary Information (SI), ZZ3_II formed an H-bond with the negatively charged Asp165 side chain at 1.9 Å and strongly interacted with this residue. Since Asp165 was included in the H6/H7 loop, which contributes to the aggregation of FtsZ, ZZ3_II was expected to change the conformation of the H6/H7 loop to inhibit the aggregations of FtsZ. Conversely, there was no additional H-bond between ZZ3_II and the FtsZ residues. Consequently, BE between FtsZ and ZZ3_II was significantly (32.2 kcal/mol) smaller than that of the pristine ZZ3.

There is no H-bond between ZZ3_III and the FtsZ residues (Figure S2), although it exhibited many electrostatic interactions with Met163, Gly164, Glu179, and Ala235 of FtsZ. Consequently, BE of ZZ3_III was 13.4 kcal/mol smaller than that of ZZ3.

As shown in Figures S3 and S4, ZZ3_IV and ZZ3_V exhibited similar binding properties with FtsZ. Both derivatives formed a strong H-bond with the Asp165 side chain and interacted electrostatically with Met163. However, the other residues were separated from the derivatives. Consequently, their BEs were smaller than that of ZZ3.

Unlike ZZ3_IV and ZZ3_V, ZZ3_VI and ZZ3_VII interacted with many FtsZ residues, as shown in Figures S5 and S6. These derivatives possessed an oxygen atom each on the A-part of ZZ3. This oxygen atom in ZZ3_VI formed an H-bond with the backbone between Ser221 and Ala222 and significantly changed the binding conformation of ZZ3_VI. In fact, the A-part was separated from the ZZ3 binding pocket of FtsZ, and the H6/H7 loop became free. ZZ3_VII exhibited a similar binding mode to that of ZZ3_VI (Figure S6). Therefore, these derivatives are not suitable inhibitors of the aggregation of FtsZ, which was caused by the interactions between the H6/H7 loops of the neighboring FtsZs.

ZZ3_VIII possessed the largest BE among the seven derivatives, and its size was almost the same as that for ZZ3. As indicated in Figure S7, ZZ3_VIII formed two H-bonds with Ser221 and Ala222, thereby increasing its BE. However, the binding conformation of ZZ3_VIII was completely different from that of ZZ3, and it did not interact with the H6/H7 loop. Therefore, it could be concluded that ZZ3_VIII was not a suitable inhibitor of the aggregation of FtsZ.

From the aforementioned results of the ZZ3 derivatives with A-part replacements, the replacement of the A-part did not exert a positive effect on the interactions between FtsZ and ZZ3. Consequently, we proposed other ZZ3 derivatives by replacing its other parts.

### 3.2. Binding Properties between FtsZ and the ZZ3 Derivatives by Replacing the B- or D-Part

**Figure 4:**
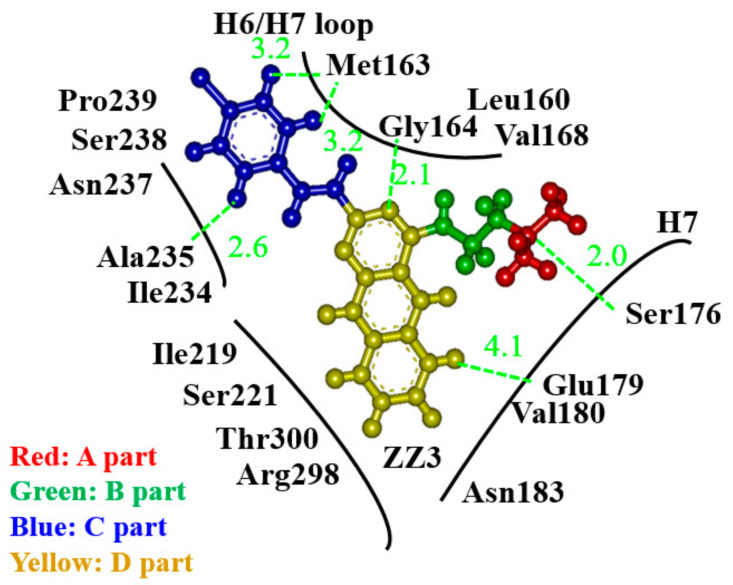
A schematic view of relative positions of FtsZ residues around ZZ3. Green lines indicate the distances (Å) between the atoms of FtsZ residue and ZZ3.

To elucidate the possibility that other replacements of ZZ3 could improve its binding affinity with FtsZ, we verified the FtsZ residues that existed around ZZ3. As shown in Figure 4, since the A-, C-, and D-parts of ZZ3 interacted with some residues of FtsZ, their replacements were not expected to enhance the binding affinity of ZZ3 to FtsZ. Accordingly, we considered the B-part and replaced it with the other group to enhance the binding of ZZ3 to FtsZ. In fact, since Leu160, Gly164, and Val168 existed near the B-part, its replacement was expected to enhance the binding between ZZ3 and these FtsZ residues (Leu160, Gly164, and Val168). Figure 4 also shows that there was no strong interaction between the left side of the D-part and the FtsZ residues. Thus, the alteration of this part was expected to enhance the binding of ZZ3 to FtsZ residues, such as Ile219, Ser221, Arg298, and Thr300, as implied in Figure 4.

**Figure 5:**
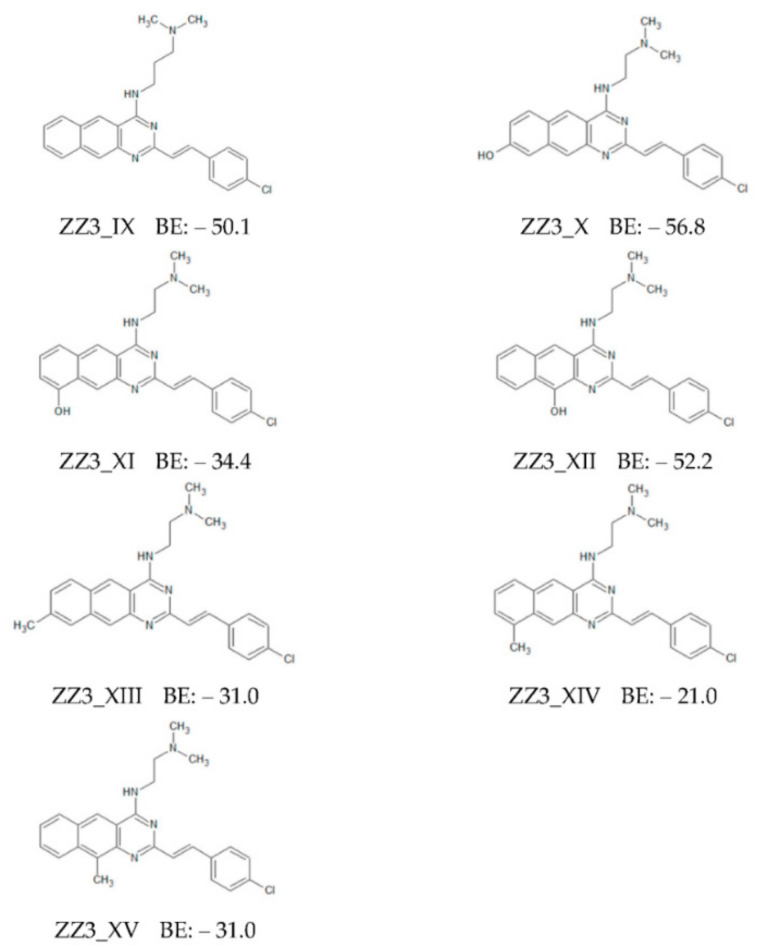
Chemical structures of ZZ3 derivatives with replacing B- or D-part, and their binding energies (BE: kcal/mol) to FtsZ evaluated by ab initio FMO method.

First, a CH_2_ group was introduced to the B-part of ZZ3 to shift ZZ3 to the left side in Figure 4 and facilitate interactions between the D-part and the FtsZ residues. This derivative was named ZZ3_IX (Figure 5). In addition, we introduced an OH group to the left side of the D-part to enhance its electrostatic interactions with the FtsZ residues. There are three possible points of introduction, which corresponded to three ZZ3 derivatives (ZZ3_X, ZZ3_XI, and ZZ3_XII). Furthermore, to enhance the hydrophobic interactions between the D-part and FtsZ residues, a CH_3_ group was introduced to different positions of the D-part to produce the ZZ3_XIII, ZZ3_XIV, and ZZ3_XV derivatives. Their chemical structures and BEs to FtsZ were evaluated by the ab initio FMO method (Figure 5).

Furthermore, we verified their chemical and pharmacokinetic properties on the SwissADME [17] and PreADMET [18] software. Furthermore, as listed in Table 1, all the ZZ3 derivatives satisfied Lipinski’s rules of five [19] and Veber’s rule, indicating that they possess chemical properties that make them suitable pharmaceutical agents.

ZZ3_IX possessed a similar BE to ZZ3 (Figure 5). The B-part of ZZ3_IX was bent significantly, and the position of ZZ3_IX in the binding pocket is almost the same as that for ZZ3 (Figure S8). Consequently, the interactions between FtsZ and ZZ3/ZZ3_IX were similar.

**Figure 6:**
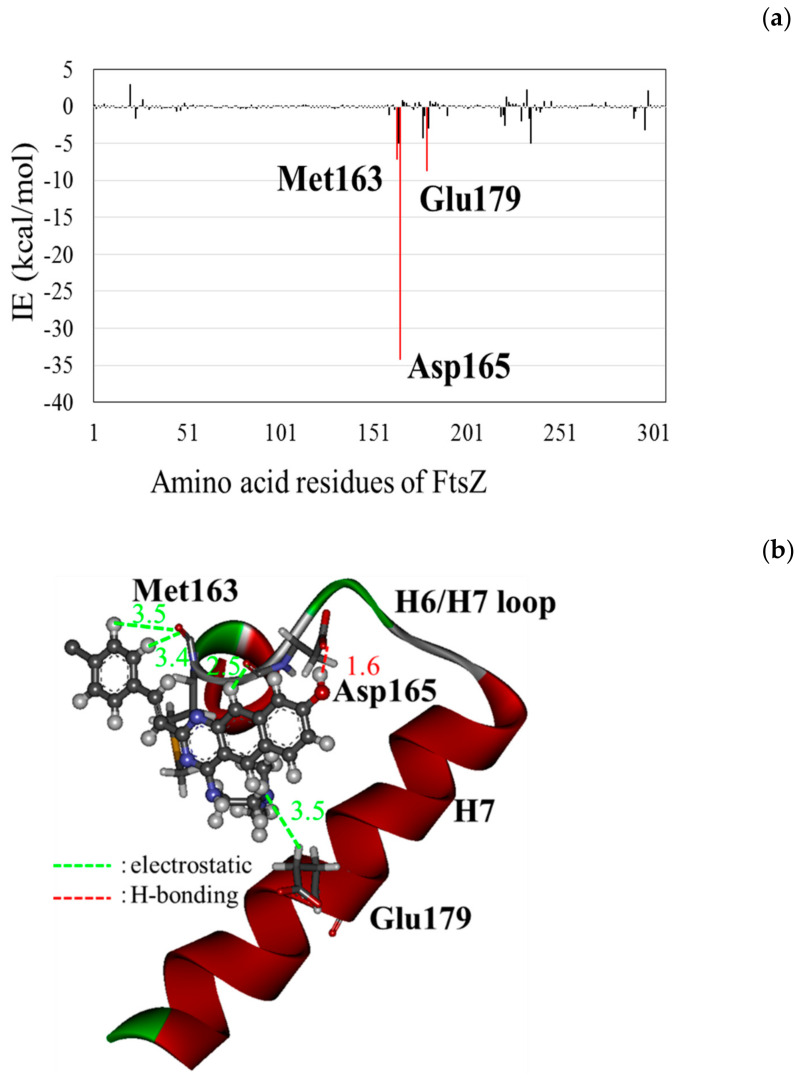
(**a**) Interaction energies (IE) and (**b**) an interacting structure between ZZ3_X (ball-and-stick model) and FtsZ residues (stick model).

Conversely, ZZ3_X possessed the largest BE among the ZZ3 derivatives proposed in this study (Figure 5). To elucidate its large BE, we investigated the interaction energies (IEs) between ZZ3_X and each of the FtsZ residues. As shown in Figure 6, ZZ3_X strongly interacted with Asp165 of FtsZ, and Met163 and Glu179 also contributed to its interaction with ZZ3_X. In fact, the OH group that was introduced to the D-part of ZZ3_X formed a strong H-bond with the Asp165 side chain at 1.6 Å. In addition, the hydrogen atom of the D-part interacted electrostatically with the main chain of Asp165. Consequently, IE between ZZ3_X and Asp165 was remarkably large (−34.3 kcal/mol). Moreover, since Asp165 was included in the H6/H7 loop, which contributed to the formations of the FtsZ aggregates and Z-ring, ZZ3_X was expected to change the loop conformation and inhibit the formation of the aggregates.

**Figure 7:**
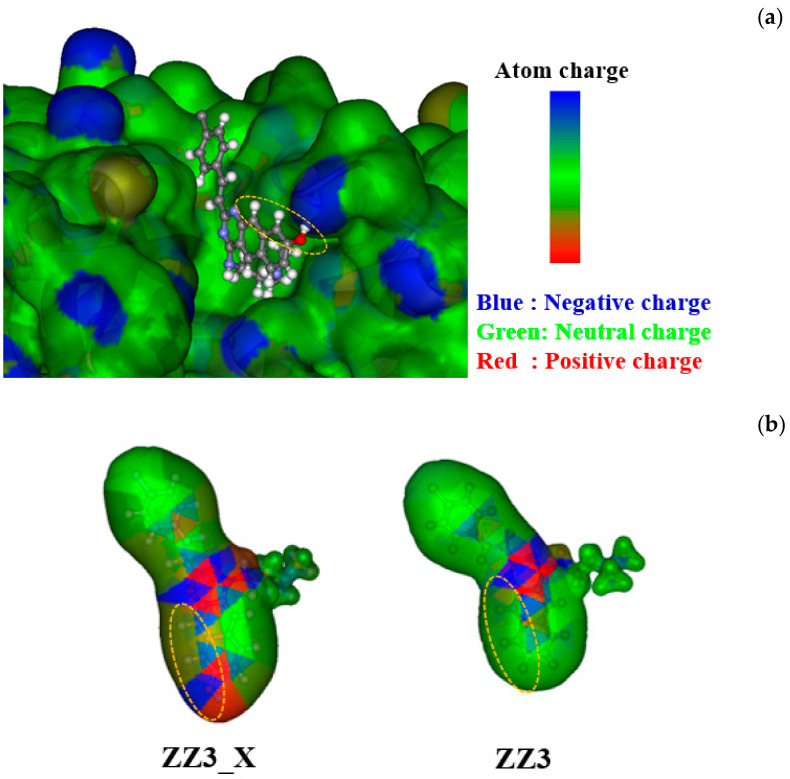
(**a**) Conformations of ZZ3 and ZZ3_X bound to the ligand-binding pocket of FtsZ, and (**b**) charge distributions for ZZ3 and ZZ3_X. The part enclosed by a yellow ellipse is a replaced part of D-part of ZZ3.

To elucidate the influence of the introduction of OH into the D-part of ZZ3, we compared the binding conformations and charge distributions of ZZ3 and ZZ3_X. As shown in Figure 7a, ZZ3 and ZZ3_X exhibited almost the same conformations in the ligand-binding pocket of FtsZ, although the part enclosed by a yellow ellipse was shifted by the introduction of an OH group to the D-part of ZZ3. This shift produced a strong H-bond between the OH group and the Asp165 side chain of FtsZ (Figure 6). The charge distribution of ZZ3 was significantly changed by the introduction of OH (Figure 7b); there was no charge polarization on the D-part of ZZ3. Contrarily, ZZ3_X exhibited large polarization that was generated by the OH group, thus forming a strong H-bond with the negatively charged Asp165 residue. As a result, ZZ3_X possessed the largest BE to FtsZ among the ZZ3 derivatives proposed here.

Notably, the position where OH was introduced to the D-part was essential for the enhancement of its binding to FtsZ. As shown in Figure 5, BEs of ZZ3_XI and ZZ3_XII with different OH positions on the D-part were smaller than that of ZZ3, indicating that the introduction of OH exerted a negative effect on the binding affinity of ZZ3 with FtsZ. To explain this effect, we investigated the specific interactions between these derivatives and the FtsZ residues. Figures S9 and S10 show that ZZ3_XI and ZZ3_XII exhibited significantly different conformations from ZZ3_X in the ligand-binding pocket of FtsZ. Therefore, these results revealed that the position of the introduced OH on the D-part of ZZ3 should be cautiously considered for the enhancement of the interaction between the ZZ3 derivative and FtsZ residues.

Further, we introduced a CH_3_ group to the D-part of ZZ3 and investigated its effect on the interactions between ZZ3 and the FtsZ residues. The chemical structures and BEs of the three derivatives (ZZ3_XIII, ZZ3_XIV, and ZZ3_XV) are shown in Figure 5. BE is significantly reduced by introducing CH_3_ to ZZ3. To explain this reduction, we investigated the interactions between the derivatives and FtsZ residues. As shown in Figures S11–S13, these derivatives could not bind to the same position as ZZ3. The introduction of a bulky CH_3_ group likely caused a steric hindrance between the D-part and residues that existed around the ligand-binding pocket of FtsZ, thus revealing that the introduction of the CH_3_ group to the D-part of ZZ3 was unsuitable for the enhancement of the binding affinity between the ZZ3 derivative and FtsZ.

As mentioned, the ZZ3_X derivative, which possesses an OH group that was introduced to the D-part of ZZ3, was confirmed to possess the highest BE to FtsZ. The OH group created the charge polarization on the D-part, which produced the strong H-bond between it and the Asp165 side chain (Figure 6). This electronic effect induced by the OH group was essential for proposing novel potent inhibitors of FtsZ, and the molecular simulations at an electronic level were employed to elucidate the effect.

## 4. Conclusions

To propose potent inhibitors of Mtb FtsZ aggregations, we designed novel derivatives based on Zantrin ZZ3, which had been confirmed to strongly bind to FtsZ in our previous molecular simulations [11], as well as by experiments [15], and the specific interactions between FtsZ and the derivatives were investigated by molecular simulations, which were based on protein–ligand docking, classical MM energy minimizations, and ab initio FMO calculations. The following features of the ZZ3 derivatives were elucidated at an electronic level.

(1) The derivative, ZZ3_X, in which an OH group was introduced in the D-part of ZZ3, possessed the largest BE to FtsZ due to the strong H-bond between the OH group and Asp165 side chain.
(2) Since Asp165 was included in the H6/H7 loop, which was beneficial for the aggregation of FtsZ, ZZ3_X was expected to change the conformation of the loop to inhibit the aggregations.
(3) The replacement of the A- and B-parts of ZZ3 did not exert any positive effect on the enhancement of the interactions between ZZ3 and FtsZ.

## Figures and Tables

**Table 1:** Chemical properties of Z3, ZZ3 and its derivatives created by SwissBioisostere [16]; molecular weight (MW, Da), number of rotatable bonds (RB), number of H-bond acceptors (HBA), number of H-bond donors (HBD), octanol-water partition coefficient (LogP), and polar surface area (PSA) evaluated by SwissADME [17].

Ligand	MW	RB	HBA	HBD	LogP	PSA
Z3	431.0	8	3	1	4.65	3.33
ZZ3	402.9	6	3	1	4.24	3.12
ZZ3_II	402.9	7	3	2	4.24	3.11
ZZ3_III	417.0	7	3	1	4.45	3.22
ZZ3_IV	388.9	6	3	2	4.04	3.01
ZZ3_V	374.9	5	3	2	3.83	2.87
ZZ3_VI	403.9	7	3	1	4.24	3.09
ZZ3_VII	389.9	6	3	1	4.04	2.96
ZZ3_VIII	375.9	5	3	2	3.83	2.82
ZZ3_IX	417.0	7	3	1	4.45	3.21
ZZ3_X	418.9	6	4	2	3.42	3.11
ZZ3_XI	418.9	6	4	2	3.42	3.09
ZZ3_XII	418.9	6	4	2	3.42	3.17
ZZ3_XIII	417.0	6	3	1	4.45	3.25
ZZ3_XIV	417.0	6	3	1	4.45	3.24
ZZ3_XV	417.0	6	3	1	4.45	3.27

**Table 2:** Pharmacokinetic properties predicted by PreADMET [18] for Z3, ZZ3 and its ZZ3 derivatives created by SwissBioisostere [16]. Blood-brain barrier (BBB) penetration is the steady-state concentration of radiolabeled compounds in brain and peripheral blood. Caco2 cell permeability (Caco2) (nm/sec) is values for evaluating intestinal absorption of drug candidates. Human intestinal absorption (HIA) is a value-predicted human intestinal absorption rate. Plasma protein binding (PPB) indicates the strength to protein. Toxicity for carcino-mouse (Mouse) and carcino-rat (Rat), and the inhibition risk of human ether-a-go-go related gene (hERG). The acceptable ranges of these properties are as follows. (1) BBB larger than 2 suggests high absorption to central nervous system (CNS), while BBB less than 0.1 indicates very low absorption. (2) Caco2 permeability (nm/sec) is larger than 70 for high permeability, while it is smaller than 4 for low permeability. (3) HIA calculated at pH 7.4 is between 0 and 20% for poor absorption, 20–70% for moderate absorption, and 70–100% for fair absorption. (4) PPB larger than 80% indicates strong binding to protein.

Ligand	BBB	Caco2	HIA	PPB	Mouse	Rat	hERG
Z3	3.5	55.6	97.1	86.7	positive	negative	medium
ZZ3	1.5	54.3	97.1	84.4	positive	negative	medium
ZZ3_II	4.2	48.2	95.9	91.9	positive	negative	medium
ZZ3_III	2.4	55.0	97.1	85.1	positive	negative	medium
ZZ3_IV	3.1	45.8	95.9	86.6	positive	negative	medium
ZZ3_V	0.5	25.8	96.3	95.8	positive	negative	medium
ZZ3_VI	1.0	53.5	97.1	91.8	positive	negative	medium
ZZ3_VII	0.6	52.0	97.1	91.1	positive	negative	medium
ZZ3_VIII	2.4	29.8	95.9	92.3	positive	negative	medium
ZZ3_IX	1.7	54.9	97.1	83.3	positive	negative	medium
ZZ3_X	2.8	37.6	96.1	84.4	negative	negative	medium
ZZ3_XI	2.8	37.6	96.1	85.3	positive	negative	medium
ZZ3_XII	2.8	37.6	96.1	85.0	positive	negative	medium
ZZ3_XIII	2.8	54.4	97.1	84.5	positive	negative	medium
ZZ3_XIV	3.1	54.4	97.1	84.2	positive	negative	medium
ZZ3_XV	2.7	54.4	97.1	84.1	positive	negative	medium

## Supplementary Materials

The following are available online at https://www.mdpi.com/2079-6382/9/12/846/s1, Figure S1: (a) Interaction energies (IE) and (b) an interacting structure between ZZ3_II and FtsZ residues, Figure S2: (a) Interaction energies (IE) and (b) an interacting structure between ZZ3_III and FtsZ residues, Figure S3: (a) Interaction energies (IE) and (b) an interacting structure between ZZ3_IV and FtsZ residues, Figure S4: (a) Interaction energies (IE) and (b) an interacting structure between ZZ3_V and FtsZ residues, Figure S5: (a) Interaction energies (IE) and (b) an interacting structure between ZZ3_VI and FtsZ residues, Figure S6: (a) Interaction energies (IE) and (b) an interacting structure between ZZ3_VII and FtsZ residues, Figure S7: (a) Interaction energies (IE) and (b) an interacting structure between ZZ3_VIII and FtsZ residues, Figure S8: (a) Interaction energies (IE) and (b) an interacting structure between ZZ3_IX and FtsZ residues, Figure S9: (a) Interaction energies (IE) and (b) an interacting structure between ZZ3_XI and FtsZ residues, Figure S10: (a) Interaction energies (IE) and (b) an interacting structure between ZZ3_XII and FtsZ residues, Figure S11: (a) Interaction energies (IE) and (b) an interacting structure between ZZ3_XIII and FtsZ residues, Figure S12: (a) Interaction energies (IE) and (b) an interacting structure between ZZ3_XIV and FtsZ residues, Figure S13: (a) Interaction energies (IE) and (b) an interacting structure between ZZ3_XV and FtsZ residues.
