# Supplementary material for: Proposal of Potent Inhibitors for a Bacterial Cell Division Protein FtsZ: Molecular Simulations Based on Molecular Docking and ab Initio Molecular Orbital Calculations
Source: Antibiotics (Basel). 2020 Nov 26;9(12):846. doi: 10.3390/antibiotics9120846 (PMC7761175; doi:10.3390/antibiotics9120846)
Supplement: Supplementary file 1 [file antibiotics-09-00846-s001.pdf]

antibiotics

Supplementary Materials

Proposal of Potent Inhibitors for a Bacterial Cell Division Protein FtsZ: Molecular Simulations Based on Molecular Docking and ab Initio Molecular Orbital Calculations

Shohei Yamamoto [1], Ryosuke Saito [1], Shunya Nakamura [1], Haruki Sogawa [1], Pavel Karpov [2], Sergey Shulga [2], Yaroslav Blume [2] and Noriyuki Kurita [1,*]

[1] Department of **Computer Science and Engineering,** Toyohashi University of Technology, Tempaku-cho, Toyohashi, Aichi 441-8580, Japan; yamamoto@klab.cs.tut.ac.jp (S.Y.); saito@klab.cs.tut.ac.jp (R.S.); nakamura@klab.cs.tut.ac.jp (S.N.); sogawaharuki@gmail.com (H.S.)

[2] Institute of Food Biotechnology and Genomics, National Academy of Sciences of Ukraine, 2a, Osypovskogo str., Kyiv-123, Kyiv 04123, Ukraine; karpov@ifbg.org.ua (P.K.); Shulga5@i.ua (S.S.); blume.yaroslav@nas.gov.ua (Y.B.)

* Correspondence: kurita@cs.tut.ac.jp; Tel.: +81-532-44-6875

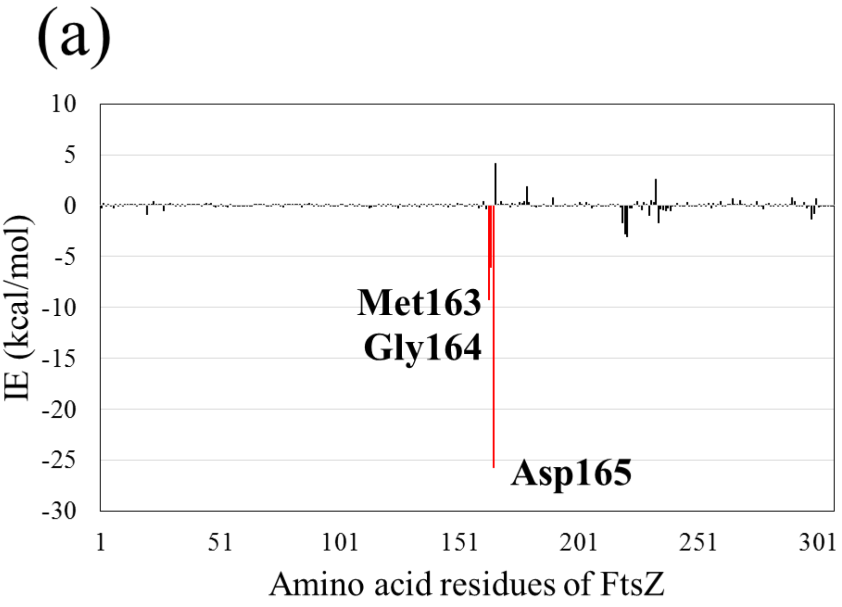

(b)

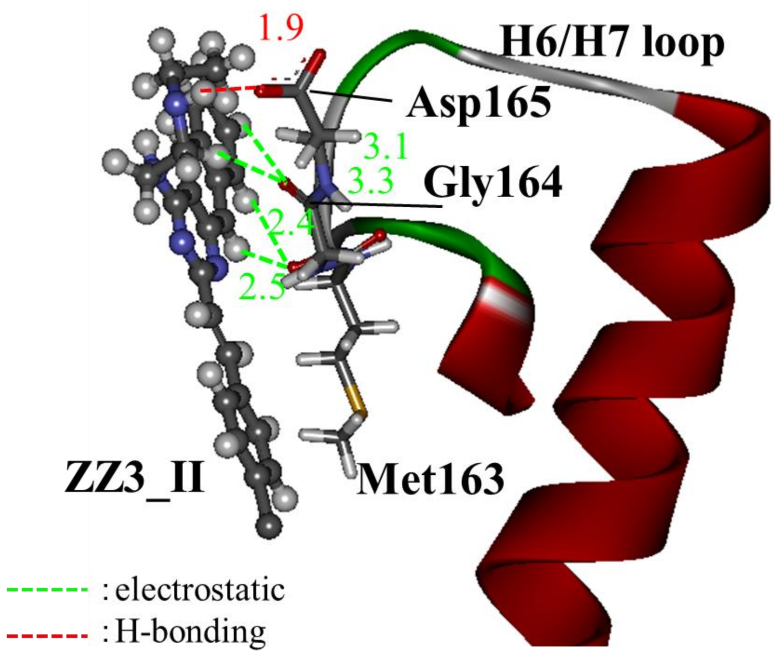

Figure S1. (**a**) Interaction energies (IE) and (**b**) an interacting structure between ZZ3_II and FtsZ residues.

(a)

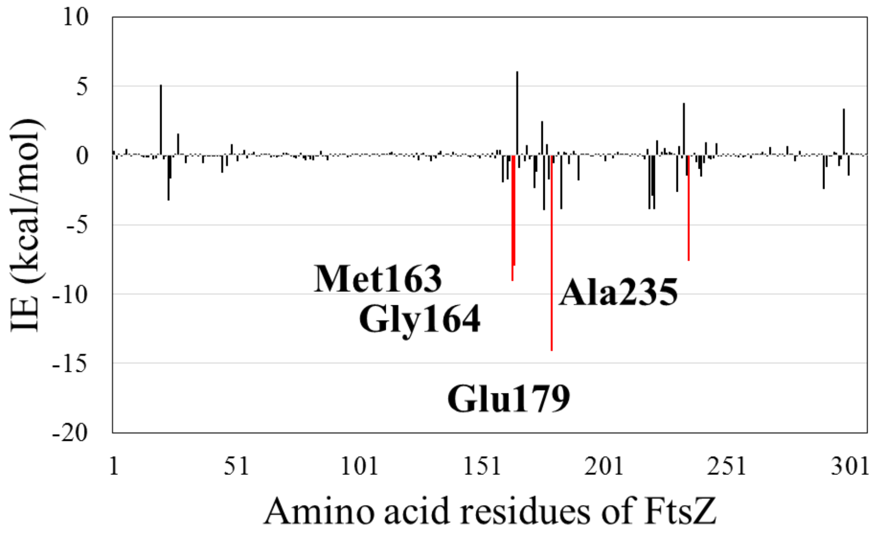

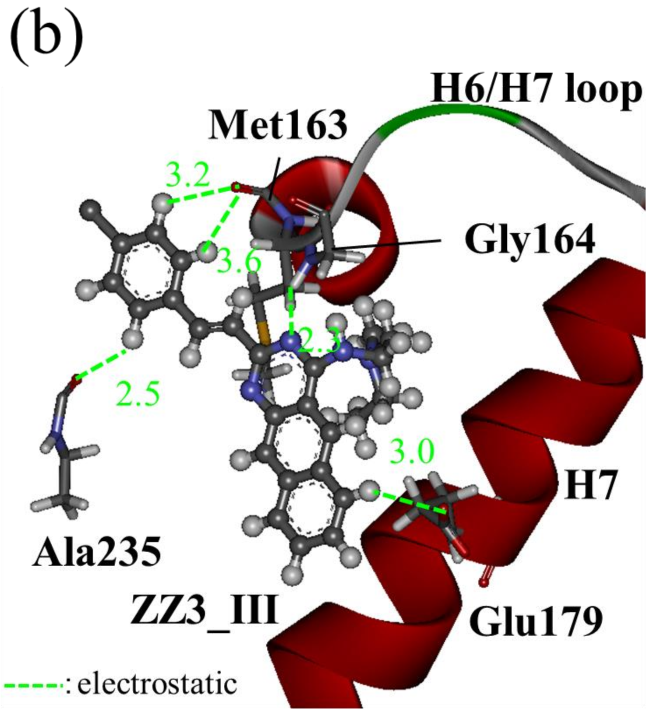

Figure S2. (**a**) Interaction energies (IE) and (**b**) an interacting structure between ZZ3_III and FtsZ residues.

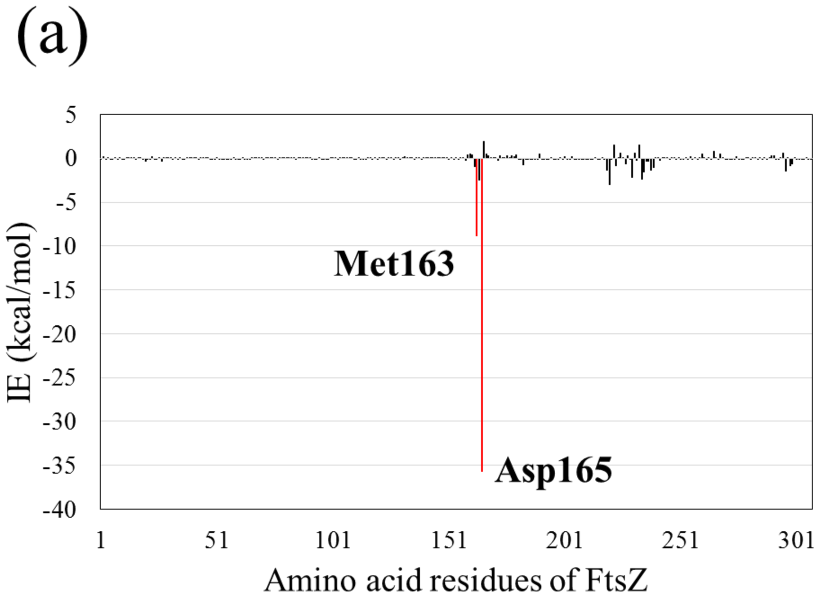

(b)

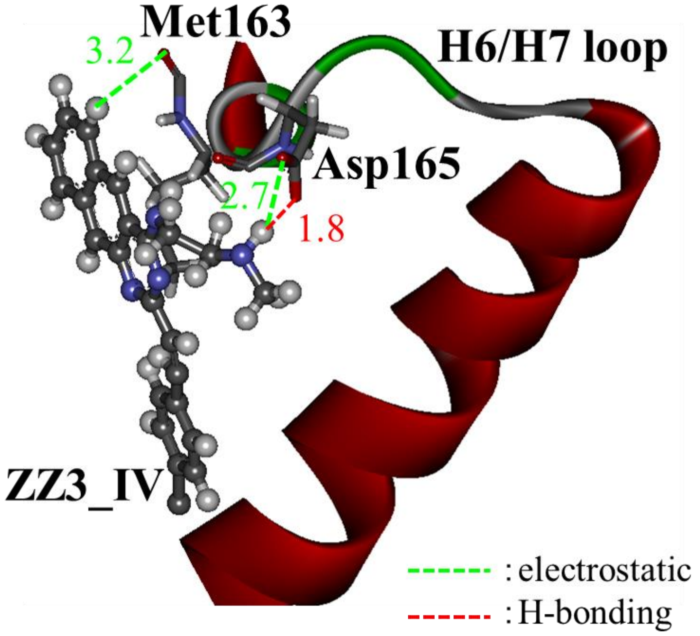

Figure S3. (**a**) Interaction energies (IE) and (**b**) an interacting structure between ZZ3_IV and FtsZ residues.

(a)

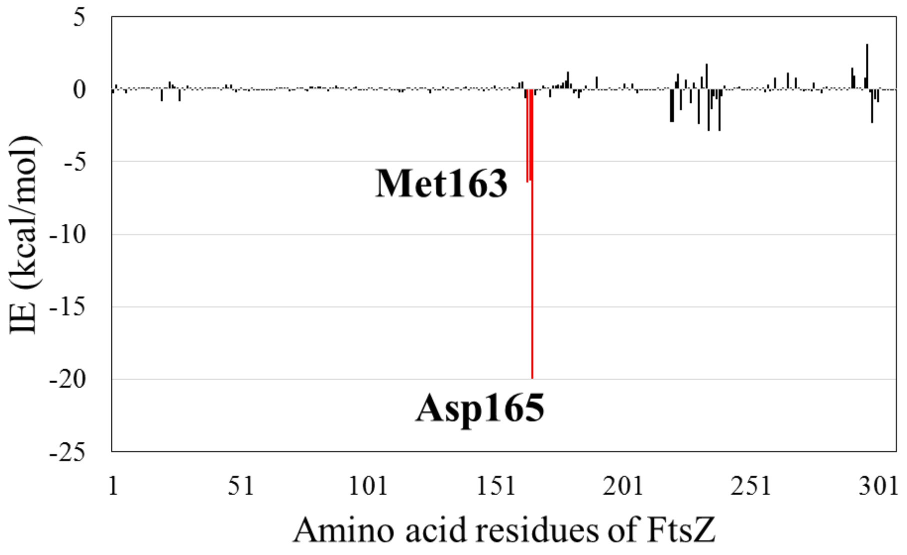

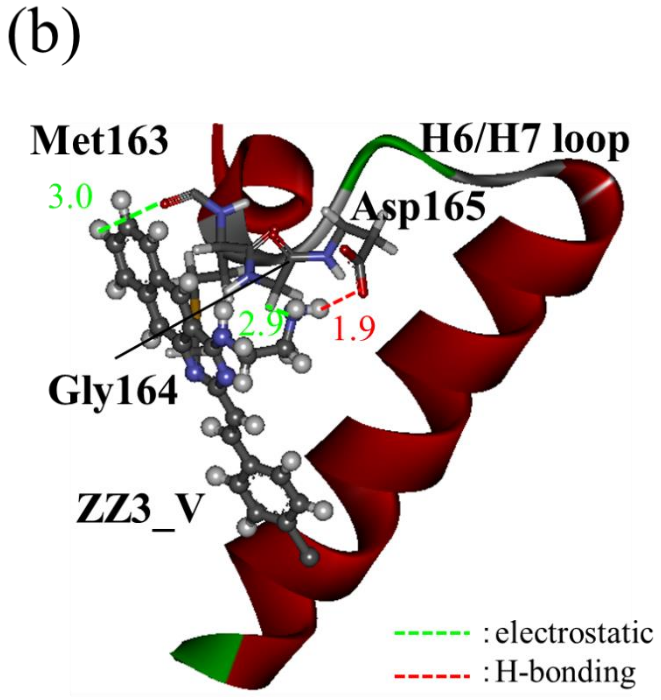

Figure S4. (**a**) Interaction energies (IE) and (**b**) an interacting structure between ZZ3_V and FtsZ residues.

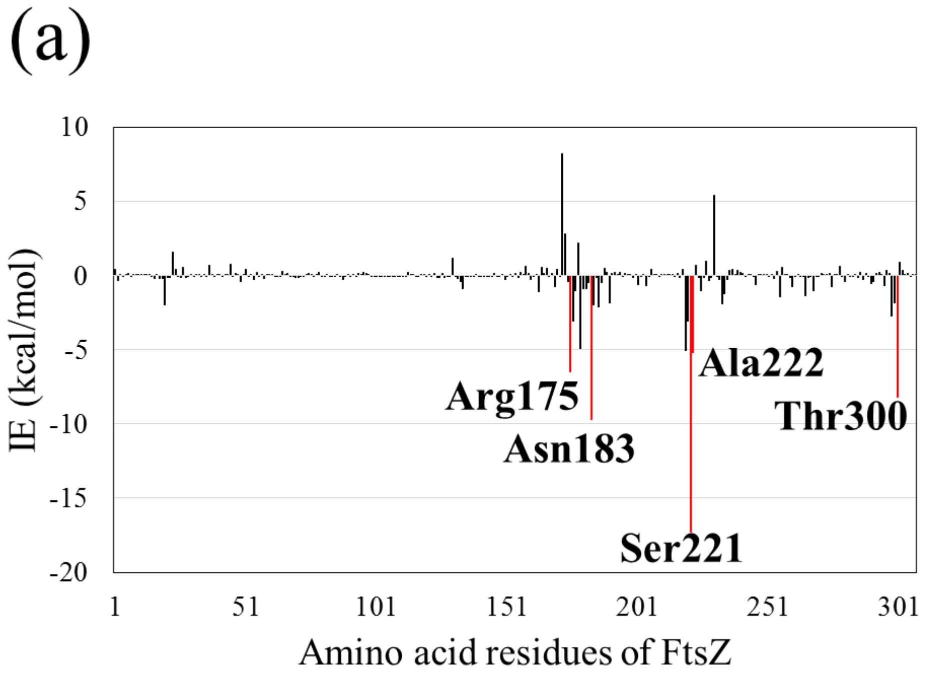

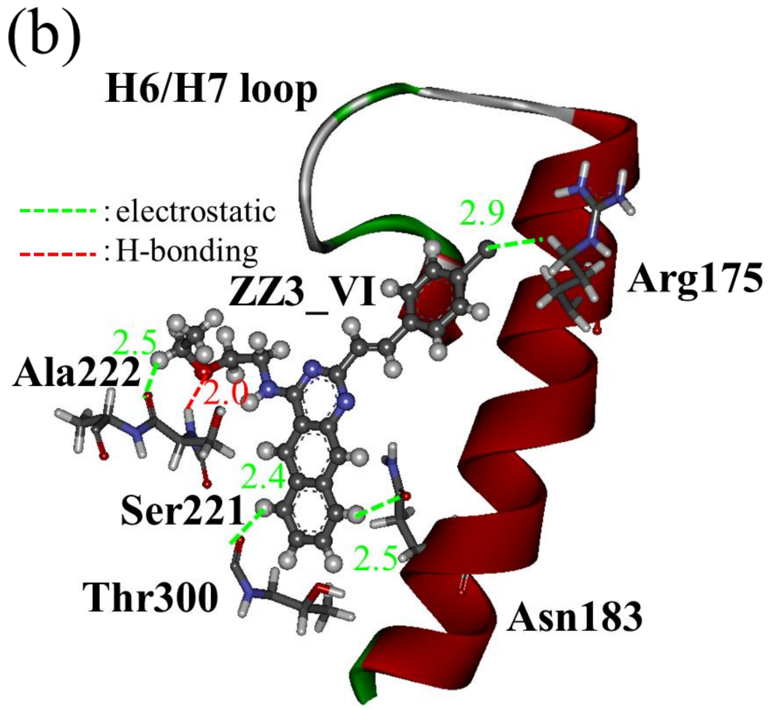

Figure S5. (**a**) Interaction energies (IE) and (**b**) an interacting structure between ZZ3_VI and FtsZ residues.

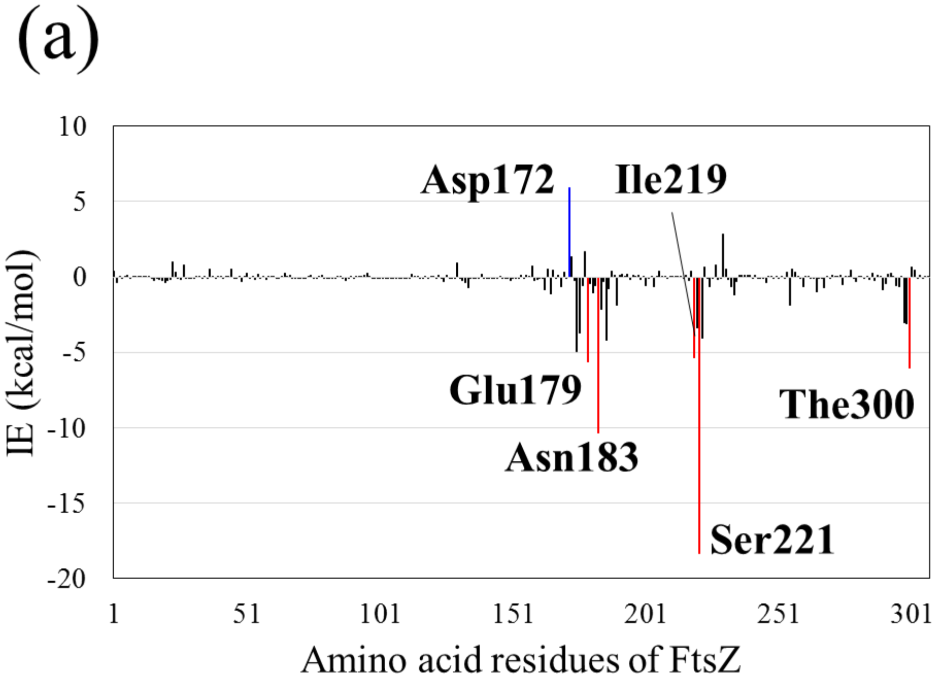

(b)

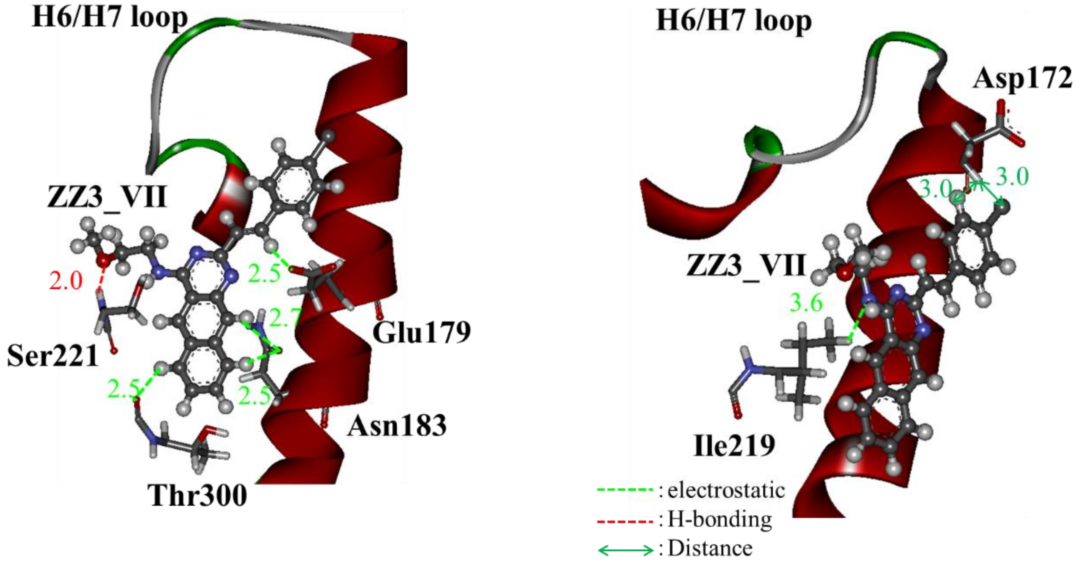

Figure S6. (**a**) Interaction energies (IE) and (**b**) interacting structures between ZZ3_VII and FtsZ residues.

(a)

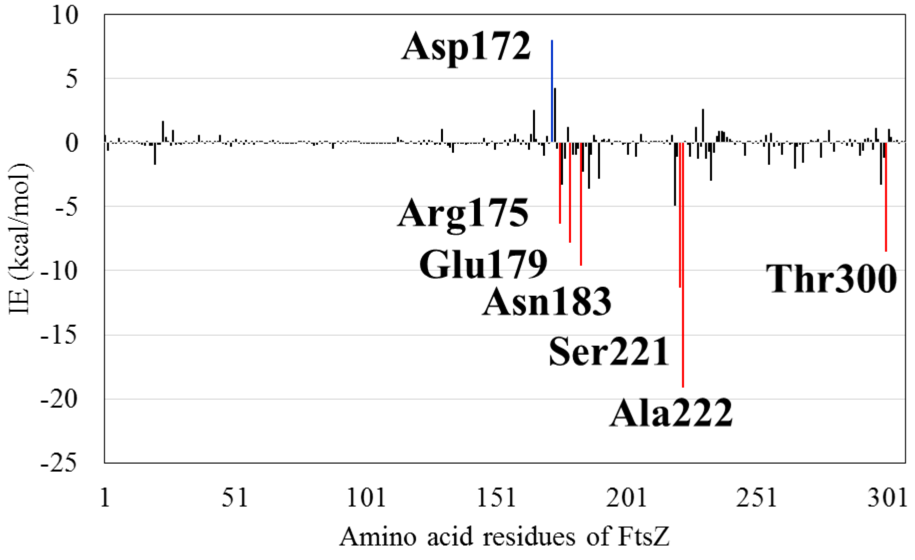

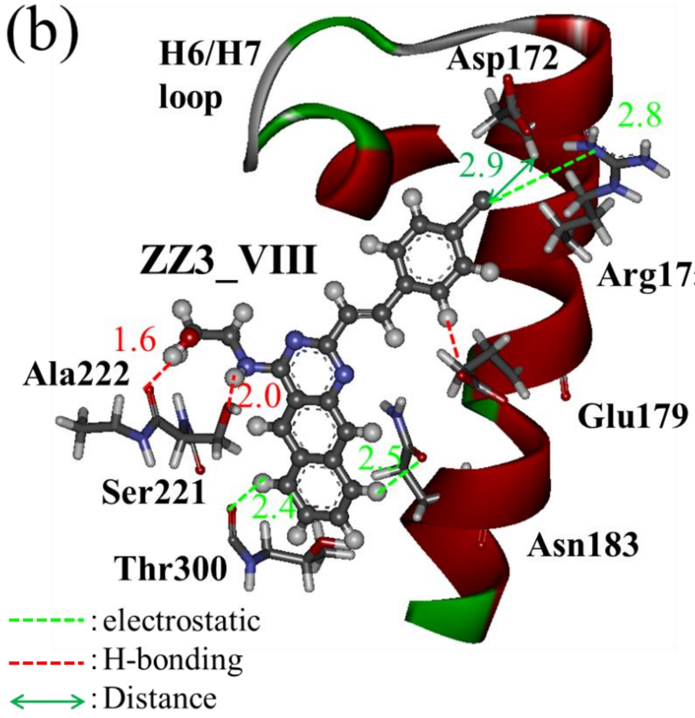

Figure S7. (**a**) Interaction energies (IE) and (**b**) an interacting structure between ZZ3_VIII and FtsZ residues.

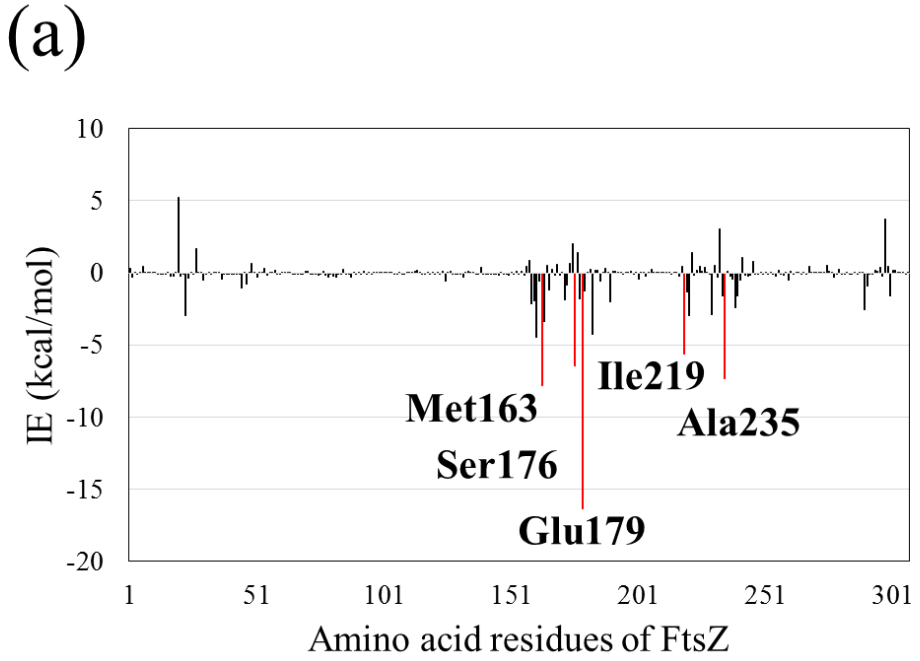

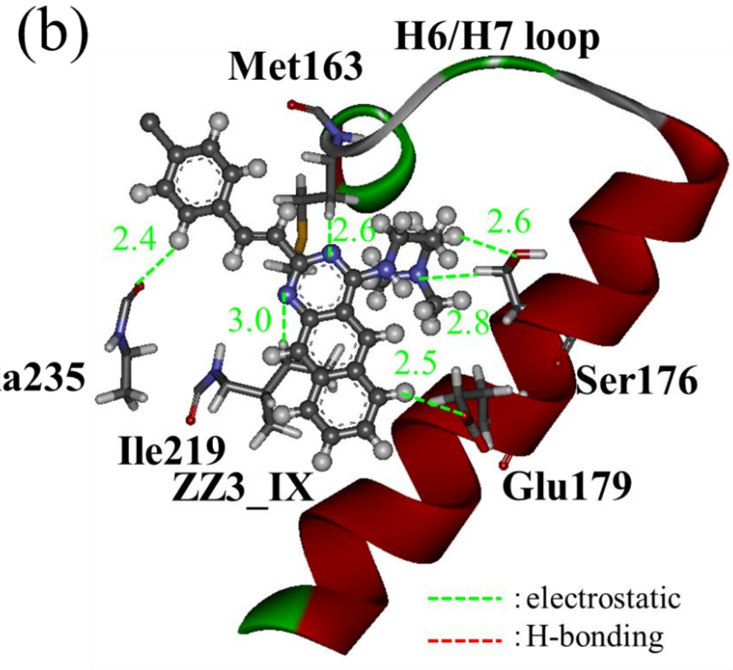

Figure S8. (**a**) Interaction energies (IE) and (**b**) an interacting structure between ZZ3_IX and FtsZ residues.

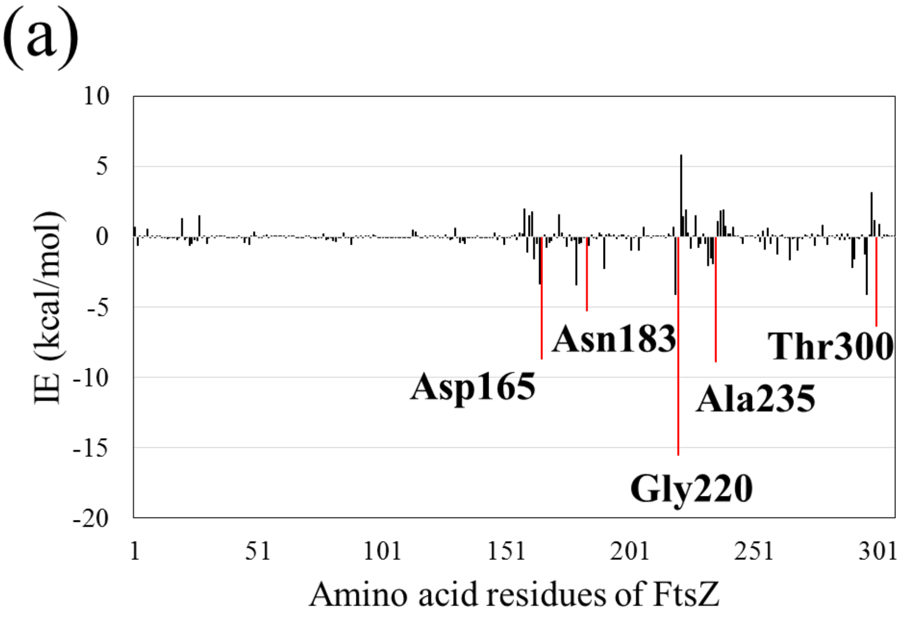

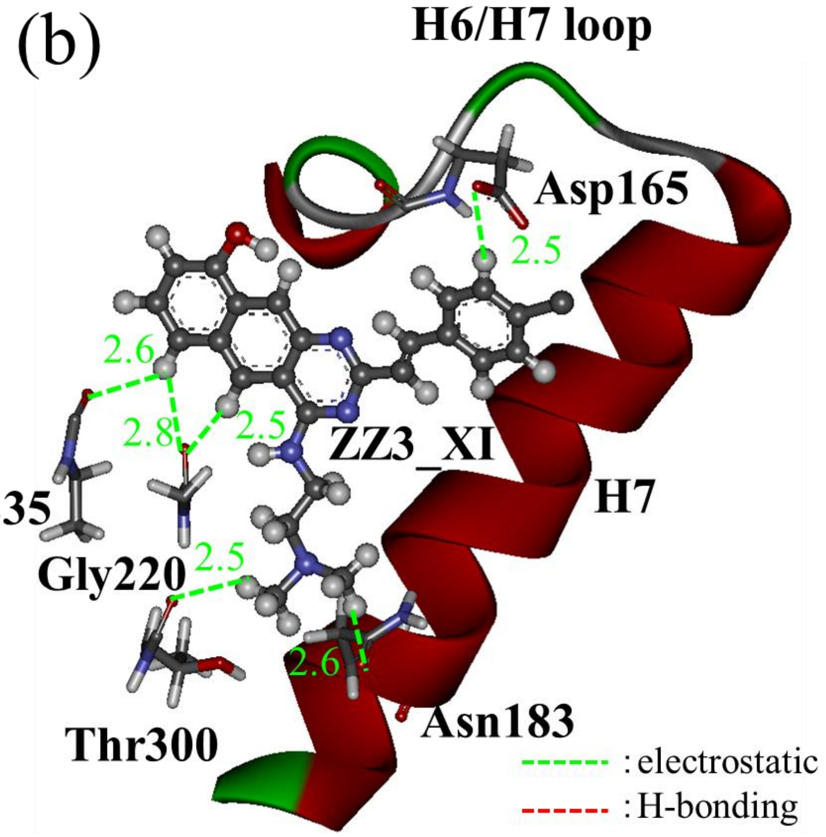

Figure S9. (**a**) Interaction energies (IE) and (**b**) an interacting structure between ZZ3_XI and FtsZ residues.

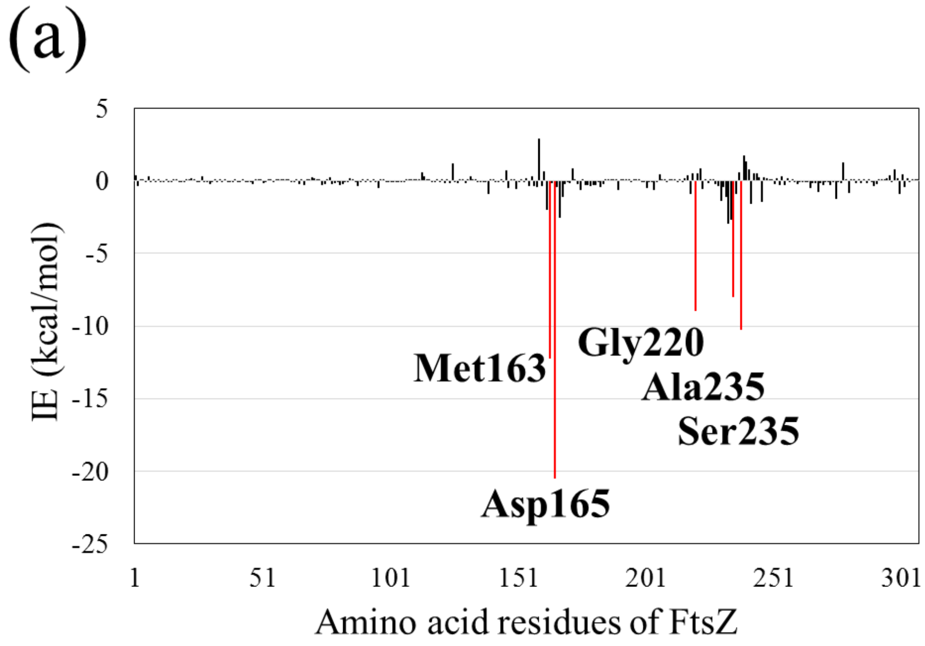

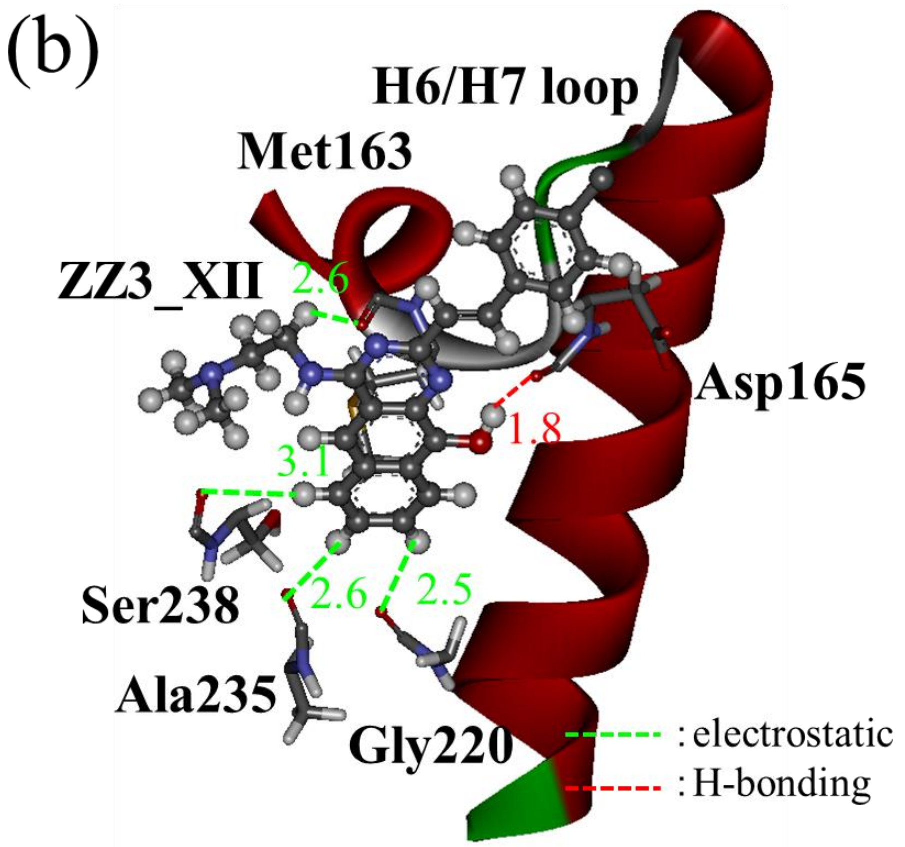

Figure S10. (**a**) Interaction energies (IE) and (**b**) an interacting structure between ZZ3_XII and FtsZ residues.

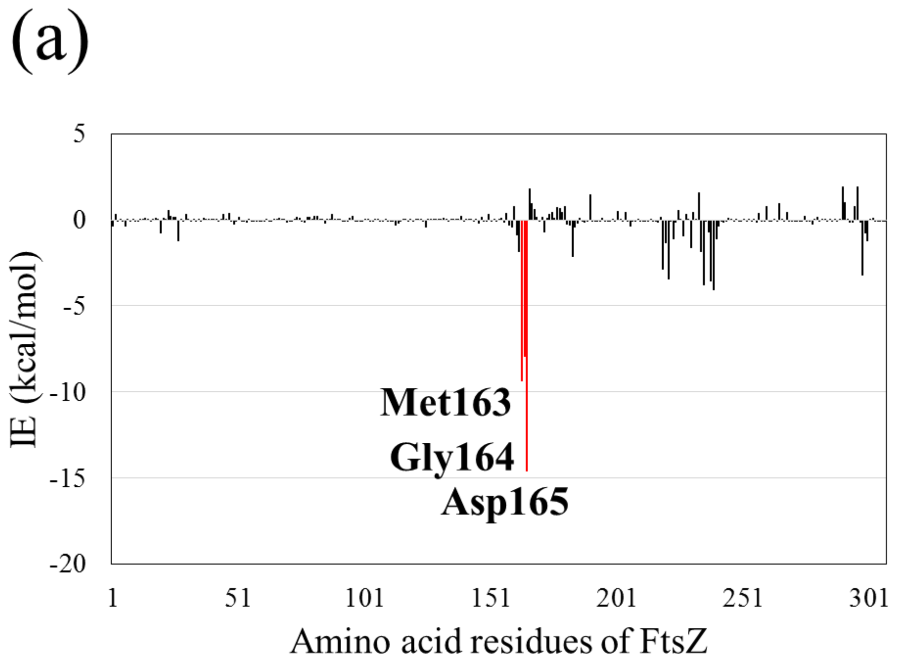

(b)

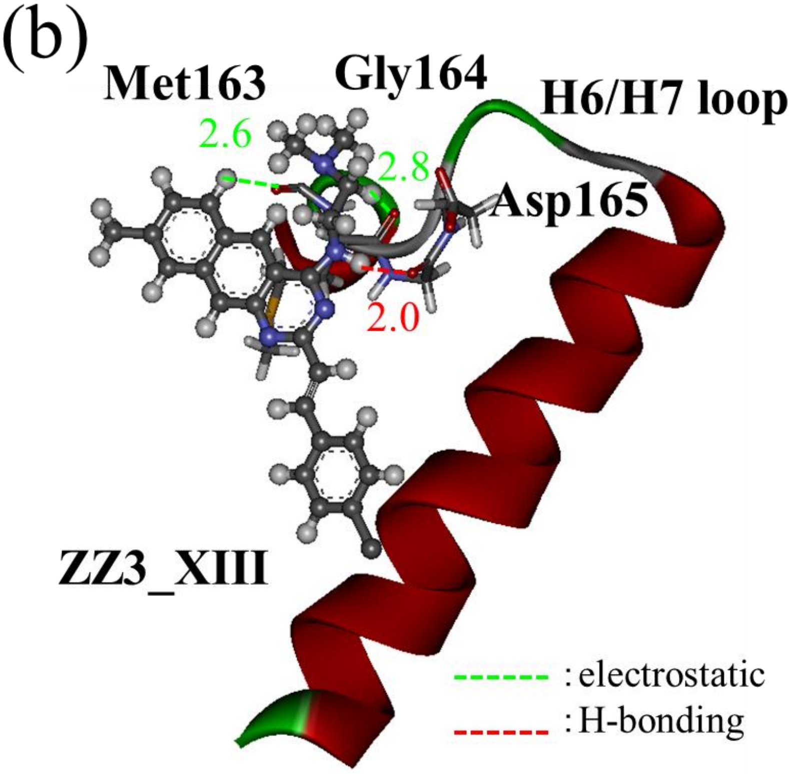

Figure S11. (**a**) Interaction energies (IE) and (**b**) an interacting structure between ZZ3_XIII and FtsZ residues.

(a)

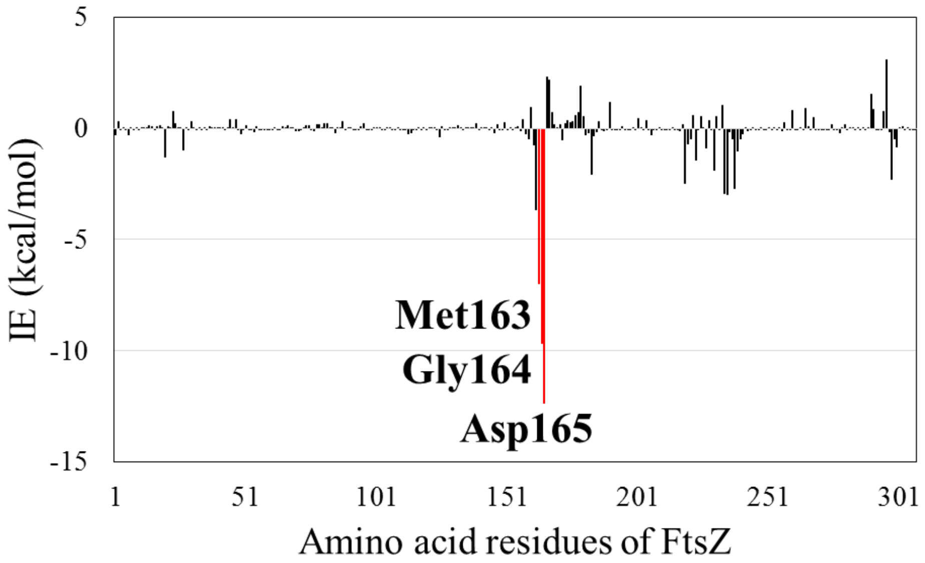

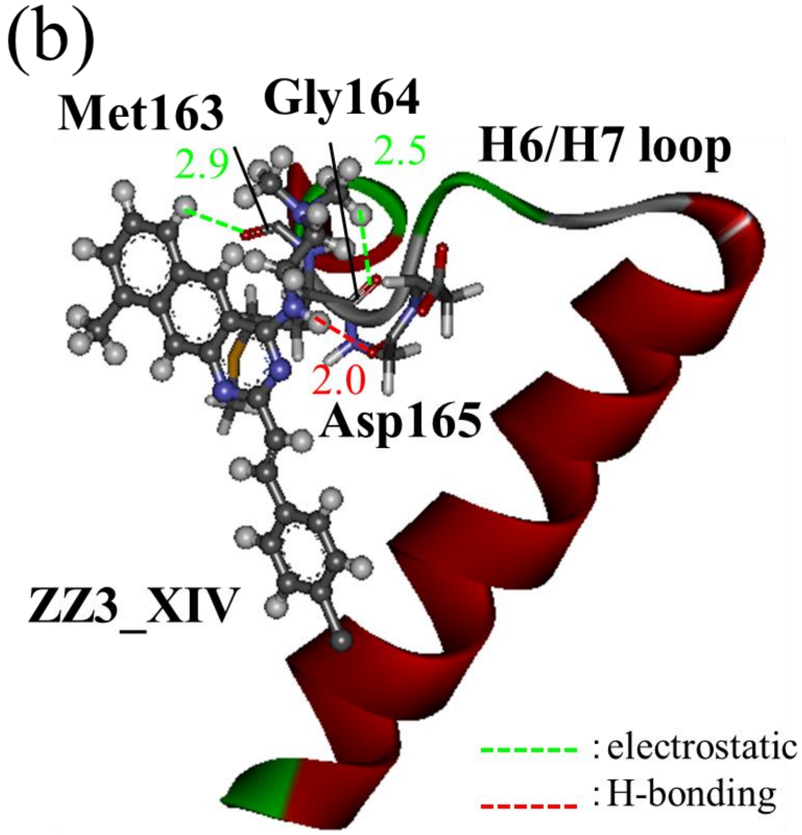

Figure S12. (**a**) Interaction energies (IE) and (**b**) an interacting structure between ZZ3_XIV and FtsZ residues.

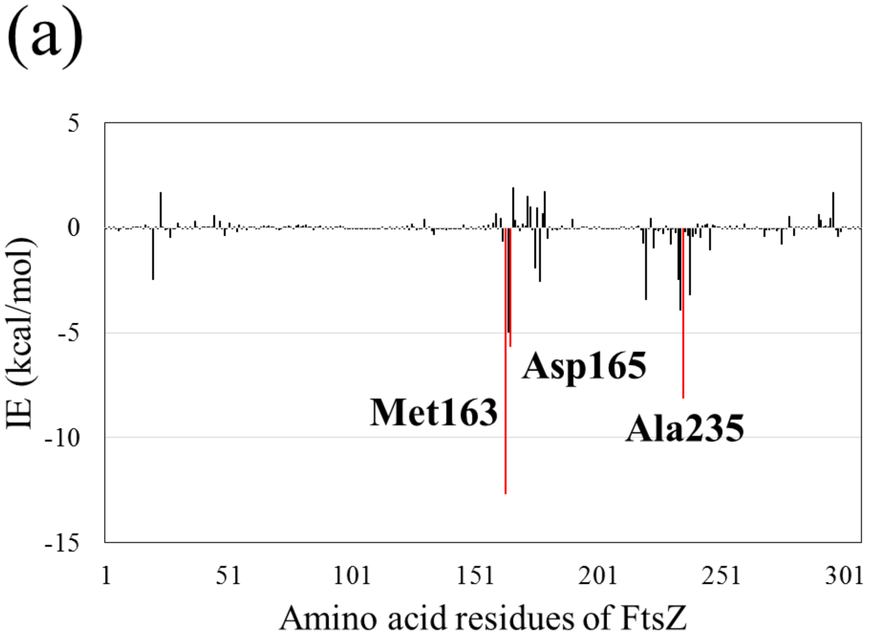

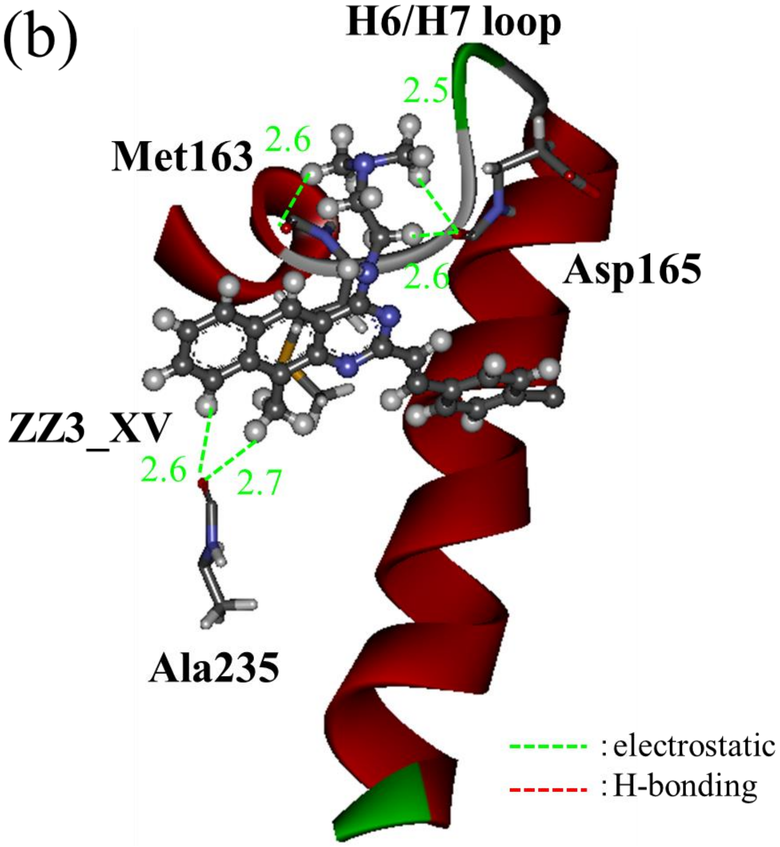

Figure S13. (**a**) Interaction energies (IE) and (**b**) an interacting structure between ZZ3_XV and FtsZ residues.
